# Rhabdomyosarcoma and Wilms tumors contain a subpopulation of noggin producing, myogenic cells immunoreactive for lens beaded filament proteins

**DOI:** 10.1371/journal.pone.0214758

**Published:** 2019-04-11

**Authors:** Jacquelyn Gerhart, Kathryn Behling, Michele Paessler, LaBraya Milton, Gregory Bramblett, Denise Garcia, Meghan Pitts, Reginald Hurtt, Mitchell Crawford, Richard Lackman, Daniela Nguyen, Joseph Infanti, Paul FitzGerald, Mindy George-Weinstein

**Affiliations:** 1 Philadelphia College of Osteopathic Medicine, Philadelphia, PA, United States of America; 2 Dept. of Biomedical Sciences, Cooper Medical School of Rowan University, Camden, NJ, United States of America; 3 Dept. of Pathology, Cooper University Health Care, Camden, NJ, United States of America; 4 Division of Hematopathology, Children’s Hospital of Philadelphia, Philadelphia, PA, United States of America; 5 Dept. of Orthopaedics, Cooper University Health Care, Camden, NJ, United States of America; 6 Lankenau Institute for Medical Research, Wynnewood, PA, United States of America; 7 Dept. of Cell Biology and Human Anatomy, University of California, Davis, Davis, CA, United States of America; Washington State University, UNITED STATES

## Abstract

Myo/Nog cells are identified by their expression of the skeletal muscle specific transcription factor MyoD and the bone morphogenetic protein inhibitor noggin, and binding of the G8 monoclonal antibody. Their release of noggin is critical for morphogenesis and skeletal myogenesis. In the adult, Myo/Nog cells are present in normal tissues, wounds and skin tumors. Myo/Nog cells in the lens give rise to myofibroblasts that synthesize skeletal muscle proteins. The purpose of this study was to screen human lens tissue, rhabdomyosarcoma cell lines, and tissue sections from rhabdomyosarcoma, Wilms and tumors lacking features of skeletal muscle for co-localization of antibodies to Myo/Nog cell markers and the lens beaded filament proteins filensin and CP49. Immunofluorescence localization experiments revealed that Myo/Nog cells of the lens bind antibodies to beaded filament proteins. Co-localization of antibodies to G8, noggin, filensin and CP49 was observed in most RC13 and a subpopulation of RD human rhabdomyosarcoma cell lines. Western blotting with beaded filament antibodies revealed bands of similar molecular weights in RC13 and murine lens cells. Human alveolar, embryonal, pleomorphic and spindle cell rhabdomyosarcomas and Wilms tumors contained a subpopulation of cells immunoreactive for G8, noggin, MyoD and beaded filaments. G8 was also co-localized with filensin mRNA. Staining for beaded filament proteins was not detected in G8 positive cells in leiomyosarcomas, squamous and basal cell carcinomas, syringocarciomas and malignant melanomas. Lens beaded filament proteins were thought to be present only in the lens. Myo/Nog-like cells immunoreactive for beaded filaments may be diagnostic of tumors related to the skeletal muscle lineage.

## Introduction

A unique lineage of myogenic cells was discovered in the epiblast of the blastocyst stage chick embryo by co-expression of the skeletal muscle specific transcription factor MyoD and bone morphogenetic protein inhibitor noggin, and binding of the G8 monoclonal antibody (mAb) [1–4]. These “Myo/Nog” cells eventually become integrated in low numbers throughout the embryo and fetus [2, 3, 5]. Regardless of their environment, Myo/Nog cells continue to express MyoD and noggin and retain the capacity to differentiate into myofibroblasts or multinucleated skeletal myofibers in response to wounding or when cultured in serum free medium, respectively [3, 5–8].

Release of noggin from Myo/Nog cells is critical for normal embryonic development [2, 3, 9]. Depletion of Myo/Nog cells within the blastocyst results in hyperactive BMP signaling, an absence of skeletal muscle, expansion of cardiac muscle, extrusion of organs through the ventral body wall and malformations of the central nervous system, face and eyes [2, 3, 9]. Ocular malformations in embryos lacking Myo/Nog cells vary in severity from anopthalmia to lens dysgenesis and overgrowth of the retina [2, 3].

Myo/Nog cells are also present in eyes of adult mice, rats and humans [7, 10, 11]. In the retina, Myo/Nog cells protect photoreceptors exposed to hypoxic stress or damaging levels of light [10, 11]. Human lens tissue contains Myo/Nog cells that surround wounds in the epithelium, synthesize skeletal muscle proteins and generate wrinkles in the underlying basement membrane [7, 8].

Myo/Nog cells also have been identified in adult skin where they are associated with hair follicles [12]. Following epidermal abrasion, Myo/Nog cells rapidly increase in number and populate the wound [12]. Additionally, Myo/Nog cells are present in skin tumors [12]. Finding Myo/Nog cells in skin tumors as well as normal tissues throughout the body led us to hypothesize that they may play a role in tumors with skeletal muscle-like properties. Rhabdomyosarcomas (RMS) exhibit histological features of skeletal muscle and express members of the MyoD family [13–15]. They are the most common soft tissue sarcoma in children [13, 14]. Multiple subtypes of RMS have been described, including embryonal (ERMS), alveolar (ARMS), pleomorphic, and spindle cell/sclerosing [13–15]. ERMS is the most common and least aggressive of the RMS tumors. ARMS tumors may arise in the extremities and trunk and are generally associated with a poorer prognosis than ERMS [13, 14]. Eighty percent of ARMS patients have a translocation of the *Pax3* or *Pax7* gene located on chromosomes 2 and 1, respectively, with the *FOXO1/FKHR* gene on chromosome 13 [16–18]. Pleiomorphic rhabdomyosarcomas are high grade, aggressive lesions with focal skeletal muscle differentiation that typically arise in the deep soft tissues of the lower limbs [19, 20]. Finally, spindle cell/sclerosing RMS represent a heterogenous group of tumors that are found in both children and adults [21].

Another type of sarcoma featuring properties of skeletal muscle is Wilms/nephroblastoma that arises in the kidneys of pediatric patients [22]. Wilms tumors are typically characterized by a triphasic appearance with an undifferentiated blastema, a fibroblast-like stroma and epithelial elements [23]. Heterologous elements sometimes seen in these tumors can resemble skeletal muscle and some cells are positive for the MyoD family member Myogenin [24]. Skeletal muscle proteins have not been detected in leiomyosarcomas that are derived from smooth muscle cells and are most commonly found in middle-aged and older adults in the retroperitoneum or extremities [20].

Leiomyosarcoma, RMS and Wilms tumors contain the intermediate filament protein desmin present in muscle [25–27]. A subclass of intermediate filament proteins, called beaded filaments (BFs), are considered to be specific to the lens [28–34]. DNA sequencing, gene mapping and immunolocalization revealed the link between the two BF proteins, CP49 (aka phakosin) and filensin (aka CP115) and the intermediate filament family [32, 33]. BFs are commonly assigned to their own class in the intermediate filament family, the Type VI Orphan filaments, because of their sequence divergence and lack of assembly into the classic 8–11 nm intermediate filaments [35]. While BFs have been demonstrated in at least five different vertebrate orders, to date these proteins have not been reported to be present in any tissue other than lens [29–33, 36].

In this report, we first document the presence of BFs in Myo/Nog cells in human lens tissue. Two RMS cell lines and tissue sections from human RMS, Wilms, leiomyosarcomas and skin tumors were then screened for Myo/Nog cell markers and BFs.

## Methods

### Human anterior lens tissue

Anterior lens tissue was removed by capsulorhexis from patients undergoing cataract surgery. The tissue was fixed in 2% paraformaldehyde within 10 minutes of its removal from the lens. This study was carried out in accordance with the Declaration of Helsinki and approved by the Main Line Health Institutional Review Board (protocol number: F/N-R11-3036L). Written informed consent was obtained from all donors.

### Culturing human rhabdomyosarcoma (RMS) cells

The human RC13 ARMS (SJCRH30 [RC13, RMS 13, SRJH30] (ATTC CRL2061) [17, 37, 38] and RD ERMS (ATCC CCL-136) [39–41] cell lines were obtained from the American Type Culture Collection (ATCC) (Manassas, VA). RC13 and RD cells were plated on tissue culture dishes and grown in RPMI medium (ATCC) and Dulbecco’s minimal essential medium (DMEM), respectively, containing 10% fetal bovine serum and 1% penicillin/streptomycin (Life Technologies, Grand Island, NY). Cells were fixed in 2% paraformaldehyde and permeabilized with 0.5% Triton X-100 prior to labeling with antibodies as described below.

### Tumor tissue sections

Paraffin embedded, 5 μm sections of tumor tissue were obtained from the Children’s Hospital of Philadelphia (CHOP), Cooper University Hospital (CUH) and US Biomax, Inc. (Rockville, MD). Single tissue sections with a width of greater than 7 mm (CHOP and CUH) and tissue microarrays (TMAs) containing sections of approximately 1 mm (CHOP and US Biomax) were utilized in this study. US Biomax’s TMAs consisted of 39 cases/75 cores (SO751), 12 cases/24 cores (SO241), 24 cases/24 cores (KD247) and 6 cases/24 cores (T0013). The CHOP TA-18 TMA contained 23 cores of normal tissue and 120 tumor cores from 40 cases. CHOP’s SRBC TMA-1 consisted of 36 cores of normal tissue and 146 tumor cores from 73 cases. Tumor tissue from was collected in accordance with the Declaration of Helsinki and approved by the Institutional Review Board of CHOP (protocol number: 13-010-191). Written informed consent was obtained from all donors.

### Immunofluorescence localization

Anterior lens tissue, cultured RMS cells and tumor tissue sections were double labeled with the G8 IgM mouse mAb [6] diluted 1:40 and a goat polyclonal antiserum to noggin (AF719; R&D Systems, Minneapolis, MN) diluted 1:100, rabbit polyclonal antisera to filensin [42] diluted 1:1000, or a mouse mAb to MyoD (MA5-12902; ThermoFisher Scientific/Invitrogen) diluted 1:100 by previously described methods [1, 2, 6]. The G8 mAb was generated by immunizing mice with chick embryo somite and segmental plate mesoderm cells [6]. Sections also were double labeled with the filensin rabbit polyclonal antiserum and a chicken polyclonal antiserum to CP49, both diluted 1:500. Chickens were immunized with human and mouse recombinant CP49 synthesized in Escherichia coli. Inclusion bodies were isolated using standard protocols, solubilized in urea and chromatographically purified. Purified antigen was provided to Aves Labs (Tigard, OR) for immunization. Reactivity and specificity of the resulting antiserum was tested by western blot, using unfractionated, homogenized mouse lens as a substrate. The unfractionated IgY fraction was used for immunochemical procedures.

The anti-human filensin 1C1 mouse monoclonal antibody was generated against human bfsp1, spanning residues 1–435, produced by bacterial expression using pET vectors and isopropyl-_-D-thiogalactopyranoside induction. Inclusion bodies were purified from bacteria using lysozyme/DNase and were subjected to high and low salt washes. Purified inclusion bodies were dissolved in 8M urea and chromatographed over a SuperDex gel filtration column using a fast protein liquid chromatography system (Amersham Biosciences). Fractions were analyzed by SDS-PAGE, and fractions enriched in the bfsp1 fragment were pooled. Mouse mAbs were produced by Antibodies Incorporated, P.O. Box 1560, Davis, CA, 95617. Initial screening of candidate clones was conducted by ELISA using extracts of human lens. Reactive clones were subsequently characterized by western blotting for reactivity against human native filensin and the recombinant fragment.

Prior to applying antibodies, sections were subjected to an antigen retrieval process involving incubation of tissue in 0.1% Triton X-100 and 0.1% sodium citrate (Sigma-Aldrich, St. Louis, MO) at 80°C. Secondary antibodies included affinity purified, F(ab´)2 goat anti-mouse IgM μ chain, goat anti-mouse IgG, donkey anti-goat IgG, goat anti-rabbit IgG and goat anti-chicken IgG conjugated with DyLight 549, Alexa 549, Alex 488 or rhodamine diluted 1:200–1:2000 depending on the lot (Jackson ImmunoResearch, West Grove, PA). Nuclei were stained with Hoechst dye 33258 (Sigma-Aldrich). Staining was analyzed with a Nikon Eclipse E800 epifluorescence microscope equipped with 4x-100x plan/APO lenses and an Evolution EO camera (MediaCybernetics). Incubation with fluorescent secondary antibodies alone did not produce punctate background fluorescence in cell cultures or tissue sections. Figures were annotated and adjusted for brightness and contrast with Adobe Photoshop CC 2014.

The percentages of single and double labeled cells in anterior lens tissue and cultured RMS cells was determined by counting the numbers of positive and negative cells in 20 consecutive fields. This sampling method was validated by comparing the percentages of labeled cells in 20 fields to that present throughout the entire tissue and culture.

### Single-molecule RNA in situ hybridization

Paired double-Z oligonucleotide probes were designed against human filensin (BFSP1) mRNA (NCBI accession number: NP_001186.1) using custom software (Advanced Cell Diagnostics, Newark, CA). *In situ* hybridization was performed by a modification of the protocol provided with the RNAscope 2.5 High Definition Reagent Kit-RED (322350, Advanced Cell Diagnostics) (Wang F et al., *J Mol Diagn*, 2012). Briefly, deparaffinized tissue sections were incubated in hydrogen peroxide, boiled in RNAscope Target Retrieval Reagents solution and incubated in RNAscope Protease Plus. Hybridization of the anti-sense probe specific for filensin mRNA occurred by step-wise incubations in AMP 1–6 solutions. Probes for the human housekeeping gene peptidylprolyl isomerase B (PPIB) and bacterial DapB were applied as positive and negative controls, respectively. Bound anti-sense probes were detected with Fast Red solutions visible with bright field optics and fluorescence in the rhodamine but not fluorescein/Alexa 488 channel. After rinsing in tap water, tissue sections were labeled with the G8 mAb and anti-IgM conjugated with Alexa 488. Images were acquired as described above.

### Western blotting

RC13 RMS cells were extracted in M-PER detergent containing Halt protease inhibitor (Thermo Scientific, Rockford, IL). Mouse lenses were homogenized in 20 mM tris buffer containing 5 mM EDTA, pelleted and resuspended in 4x sodium dodecyl sulfate polyacrylamide gel electrophoresis (SDS-PAGE) lysis buffer. RMS and lens extracts were transferred to Hybond-C Extra membrane (Amersham Biosciences, UK) following SDS-PAGE. Western blotting was performed with affinity purified, rabbit antisera raised against recombinant human filensin and a 1:1 mixture of recombinant mouse and human CP49 [42, 43]. Collection of mouse tissue was carried out in strict accordance with the recommendations in the Guide for the Care and Use of Laboratory Animals of the National Institutes of Health. The protocol was approved by the Committee on the Ethics of Animal Experiments of the University of California, Davis (Protocol Number: 19710).

### Statistical analyses

Percentages of G8+, noggin+, filensin+ and cp49+ cells in RD and RC13 RMS cell lines were analyzed for normal distribution by the Shapiro-Wilk test. Significant differences between the two cell lines for normally distributed percentages of positive cells were calculated by the T-test. Comparisons of the non-normally distributed G8+ cells in the cell lines were carried out with the non-parametric Kruskall Wallace test. Comparison of the number of subjects with G8+/noggin+, G8+/filensin+, G8+/cp49+ and filensin+/cp49+ cells in tumor sections was carried out for tumor types with ≥ 5 subjects using the Chi-square test. Pairwise comparison within significant Chi-square groups was performed using the Fisher exact test. Significant differences/p values were calculated with a 95% confidence interval. Statistical analyses were performed using Sigmaplot version 14.0.3.192.

## Results

### Antibodies to BF proteins bind Myo/Nog cells in human lens tissue

**Fig 1:**
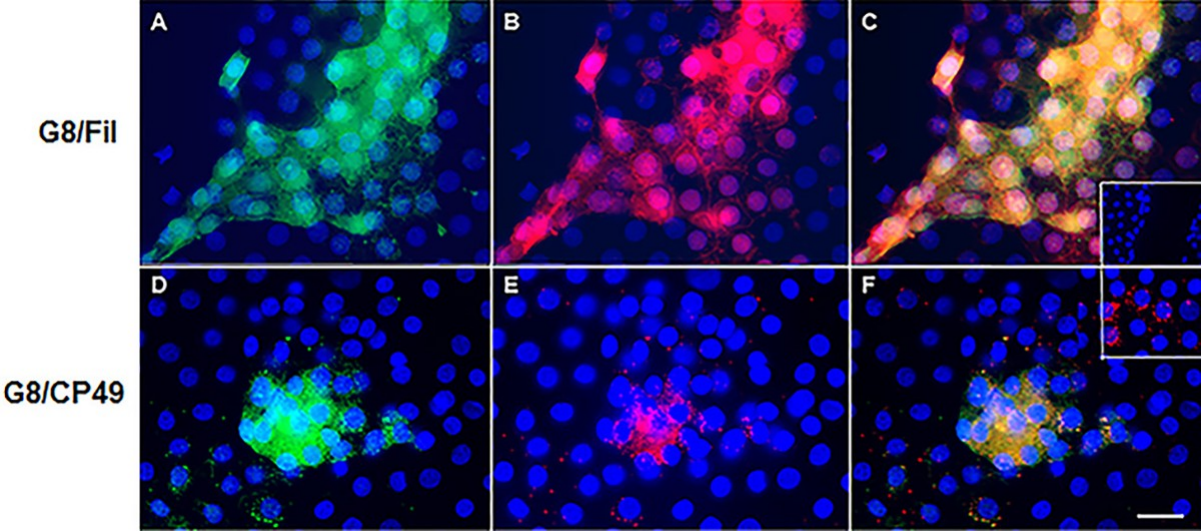
Co-localization of G8, filensin and CP49 antibodies in human lens tissue. Anterior lens tissue was double labeled with the G8 mAb (green in A and D) and polyclonal antibodies to filensin (Fil) or CP49 (red in B and E). Nuclei were stained with Hoechst dye (blue). Overlap of green and red appears yellow in merged images (C and F). The subpopulation of G8+ cells bound antibodies to beaded filament proteins (C and F). The inset in F shows G8+/CP49- cells. Lens tissue double labeled with the anti-mouse IgM and anti-rabbit Ig secondary antibodies alone were unstained (inset in C). Bar = 9 μm.

Myo/Nog cells in human lens tissue express noggin and skeletal muscle proteins, including MyoD, sarcomeric myosin and skeletal troponin T [7]. In order to assess whether Myo/Nog cells also synthesize BF proteins that historically are considered reliable and specific markers of differentiated lens fiber cells [28, 34, 36, 44], double labeling was performed with the Myo/Nog cell specific G8 mAb and antibodies to CP49 and filensin. Myo/Nog cells are present in low numbers throughout lens tissue, but are concentrated around wounds in the epithelium [7, 8]. Most G8-positive (+) Myo/Nog cells bound the filensin antibody (Table 1; Fig 1A–1C), whereas only a small and variable subpopulation of G8+ cells was labeled with the CP49 antibody (Table 1; Fig 1D–1F). As expected, antibodies to CP49 and filensin labeled a minor population of G8-negative (-) lens epithelial cells in anterior lens tissue fixed within 10 minutes of capsulorhexis (Table 1; Fig 1). This finding is consistent with the undifferentiated state of lens epithelial cells since BF proteins accumulate during their transition to lens fiber cells [36].

**Table 1:** Human lens tissue contains cells immunoreactive for G8, filensin and CP49.

Antibody	Percent
G8+	4 ± 2
Filensin+	5 ± 2
G8+ with Filensin	89 ± 10
Filensin+ with G8	52 ± 39
G8+	3 ± 1
CP49+	2 ± 2
G8+ with CP49	26 ± 34
CP49+ with G8	8 ± 12

Anterior lens tissue was fixed within 10 minutes of capsulorhexis and double labeled with the G8 mAb and polyclonal antibodies to either filensin or CP49, and species specific fluorescent secondary antibodies. % G8+, Filensin+ or CP49+ cells = (number of fluorescent cells ÷ total number of cells in a minimum of 20 fields) X 100. Percent G8+ with filensin or CP49 = (number of G8 positive cells co-labeled with the other antibody ÷ total number of G8 positive cells) X 100. % Filensin+ or CP49+ with G8 = (number of filensin+ or CP49+ cells co-labeled with G8 ÷ total filensin+ or CP49+ cells) X 100. Data represents the mean ± standard deviation of four lens explants for each pair of antibodies.

### Antibodies to Myo/Nog cells and BF proteins bind to subpopulations of RMS cells *in vitro*

**Fig 2:**
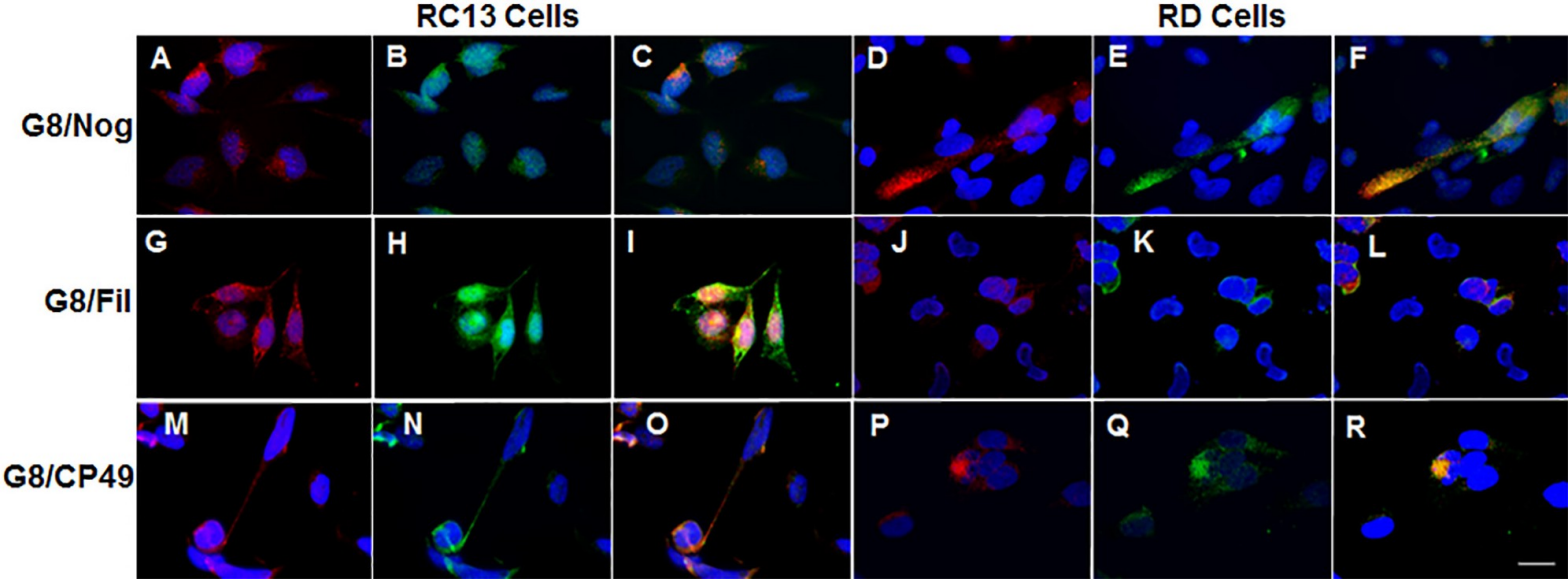
Co-localization of antibodies to Myo/Nog cell markers and beaded filament proteins in human RMS cultures. Cultures of RC13 ARMS (A-C, G-I and M-O) and RD ERMS cells (D-F, J-L and P-R) were double labeled with the G8 mAb (red) and polyclonal antibodies to noggin (Nog), filensin (Fil) or CP49 (green). Nuclei were stained with Hoechst dye (blue). Overlap of red and green appears yellow in merged images (C, F, I, L, O and R). Most RC13 ARMS cells and a subpopulation of RD ERMS cells bound antibodies to G8, noggin, filensin and CP49. Bar = 9 μm.

To further explore the apparent presence of lens proteins in human myogenic cells, double labeling was performed with antibodies to G8, noggin and BF antibodies in cultures of human RMS cells. Two cell lines were utilized in this study: 1) RC13 cells that contain the t(2;13)(q35;q14) PAX3/FOXO1 translocation found in the alveolar RMS subtype [16, 18, 45], and 2) RD cells established from an embryonal RMS tumor. Most RC13 cells bound the G8 mAb, whereas only a small subpopulation of RD ERMS cells was positive for G8 (Table 2; Fig 2). Significantly more G8+, noggin+, filensin+ and CP49+ cells were present in RC13 than RD cultures. The majority of G8+ cells in cultures of both cell lines were co-labeled with antibodies to noggin, filensin and CP49 (Table 2; Fig 2).

**Table 2:** Antibodies to G8, noggin and BF proteins label subpopulations in RMS cell lines.

	RD RMS Cells	RC13 RMS cells
% G8+	12 ± 9 (n = 35)	90 ± 10 (n = 22)
% noggin+	8 ± 4 (n = 7)	100 (n = 4)
% G8+ with noggin	98 ± 2 (n = 5)	100 (n = 4)
% noggin+ with G8	98 ± 2 (n = 5)	100 (n = 4)
% filensin+	22 ± 54 (n = 8)	91 ± 10 (n = 4)
% G8 with filensin	97 ± 5 (n = 8)	96 ± 6 (n = 4)
% filensin+ with G8	95 ± 10 (n = 8)	99 ± 2 (n = 4)
% CP49	21 ± 9 (n = 4)	88 ± 10 (n = 4)
% G8 with CP49	74 ± 21 (n = 4)	94 ± 5 (n = 4)
% CP49+ with G8	94 ± 8 (n = 4)	89 ± 13 (n = 4)

RD and RC13 RMS cells were double labeled with the G8 mAb and polyclonal antibodies to either noggin, filensin or CP49, and species specific fluorescent secondary antibodies. % G8+, noggin+, filensin+ or CP49+ cells = (number of fluorescent cells ÷ total number of cells in a minimum of 20 fields) X 100. Percent G8+ with noggin, filensin or CP49 = (number of G8 positive cells co-labeled with the other antibody ÷ total number of G8 positive cells) X 100. % Filensin+ or CP49+ with G8 = (number of filensin+ or CP49+ cells co-labeled with G8 ÷ total filensin+ or CP49+ cells) X 100. Data represents the mean ± standard deviation. The number of cultures (n) is indicated in parenthesis. The Shapiro-Wilk test demonstrated that the percentages of noggin+, filensin+ and CP49+ cells, but not G8+ cells, were normally distributed in both cell cultures. The T-test revealed significant differences between RD and RC13 cells for the percentages of noggin+ (p < 0.0001), filensin+ (p = 0.03) and CP49+ cells (p < 0.0001). The Kruskal-Wallace test showed that the percentages of G8+ cells in the RD and RC13 cell cultures were significantly different (p < 0.00001).

### BF antibodies bind proteins of similar molecular weight in RC13 RMS and lens cells

**Fig 3:**
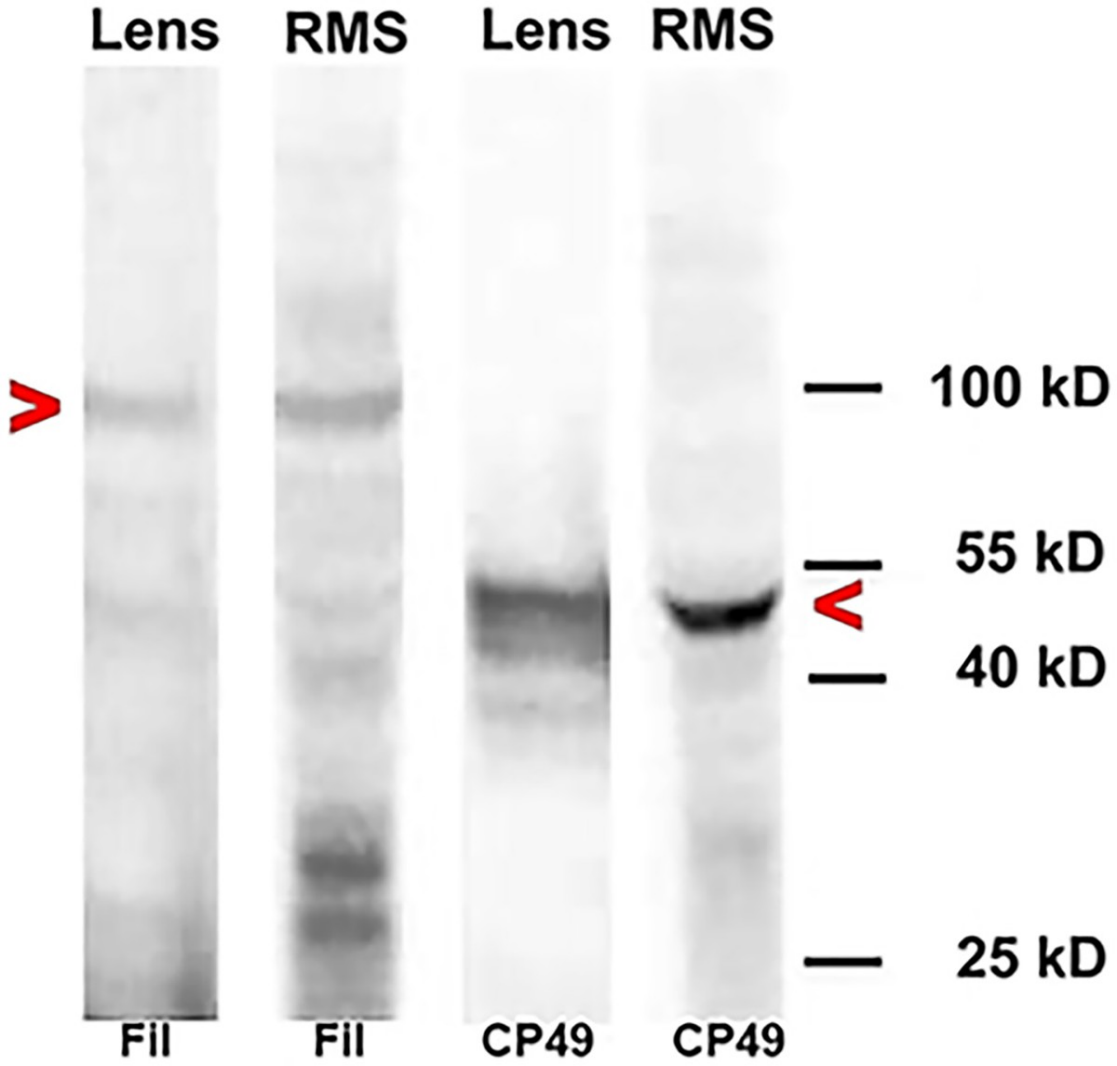
Western blot analysis of beaded filaments in RC13 RMS cells. Transblots of proteins extracted from RC13 human RMS cells and mouse lens tissue were probed with polyclonal antibodies to filensin (Fil) and CP49. Beaded filament antibodies bound proteins of similar molecular weights in RMS and lens cells.

Western blotting was performed to further validate the immunofluorescence data suggesting the presence of lens BF proteins in RC13 RMS cells. CP49 and filensin antibodies were immunoreactive with proteins of similar molecular weights in RC13 RMS and murine lens cell extracts (Fig 3). In addition to the bands of approximately 95 and 49 kD corresponding to the parent filensin and CP49 proteins, respectively, the antibodies reacted with lower molecular weight proteins. Multiple breakdown products of BF proteins are a common feature in western blots, even in native lens extracts in which aggressive measures are taken to inhibit proteolysis. That these breakdown products are derived from the parent proteins has been demonstrated using panels of monoclonal antibodies, as well as lenses from CP49 and filensin knockout mice [42, 43]. These western blotting results suggest that the epitopes of the beaded filament protein antibodies are similar in lens and RMS cells, and the RMS cell lines synthesize BF proteins.

### Myo/Nog-like cells are present in human RMS and Wilms tumors

**Fig 4:**
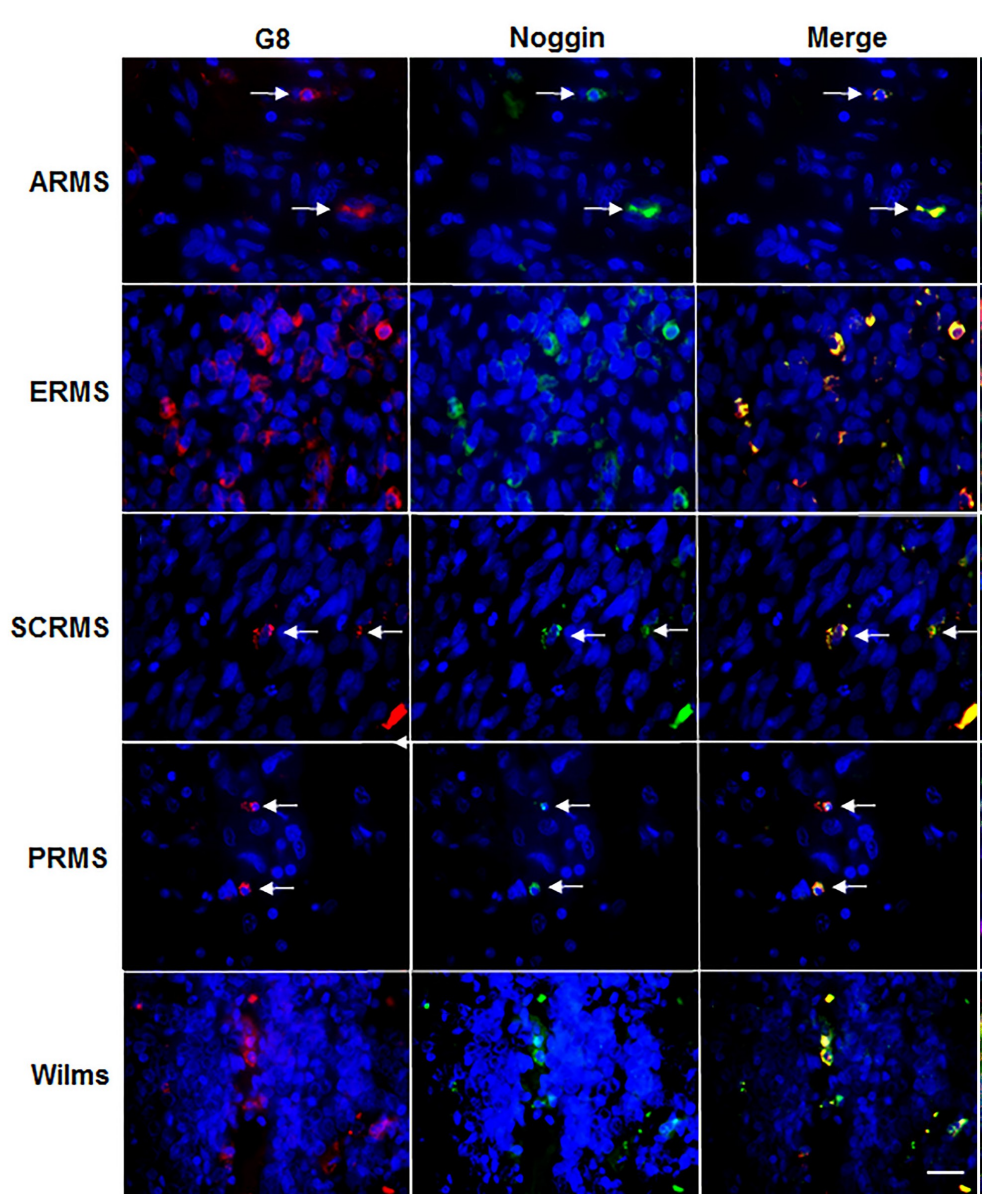
Co-localization of antibodies to G8 and noggin in human RMS and Wilms tumor tissue sections. Sections of human ARMS from striated muscle, ERMS from the retroperitoneum, spindle cell RMS (SCRMS) from the testis, pleomorphic RMS (PRMS) from fibrous tissue and Wilms (kidney) tumors were double labeled with antibodies to G8 (red) and noggin (green). Nuclei were stained with Hoechst dye (blue). Overlap of red and green appears yellow in merged images. RMS and Wilms tumors sections contain subpopulations of cells double labeled with antibodies to G8 and noggin. Bar = 9 μM.

**Fig 5:**
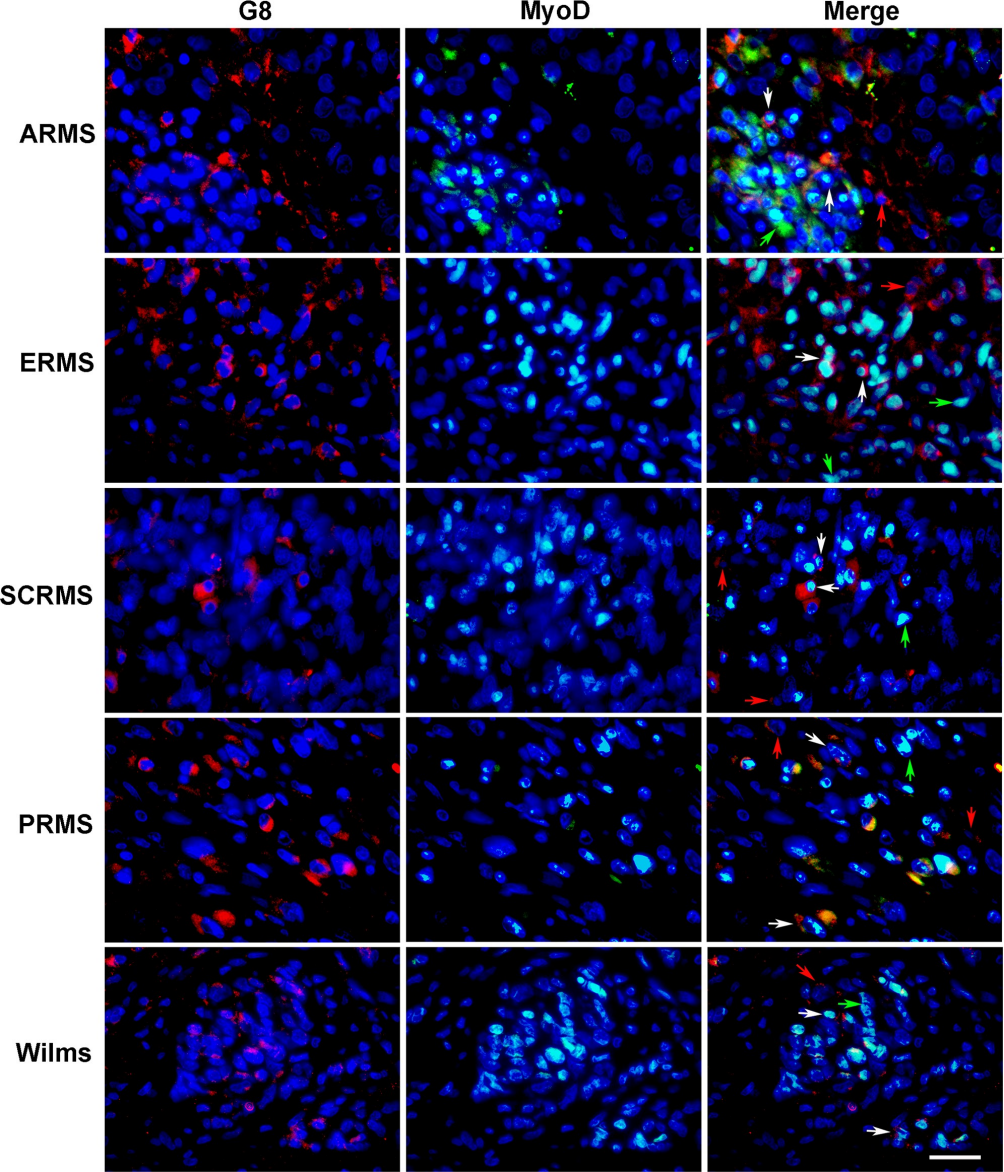
Co-localization of antibodies to G8 and MyoD in human RMS and Wilms tumor tissue sections. Sections of human ARMS from soft tissue, ERMS from the retroperitoneum, spindle cell RMS (SCRMS) from the testis, pleomorphic RMS (PRMS) from striated muscle and Wilms (kidney) tumors were double labeled with antibodies to G8 (red) and MyoD (green). Nuclei were stained with Hoechst dye (blue). Overlap of red and green appears yellow in merged images. RMS and Wilms tumors sections contain subpopulations of G8+/MyoD- cells (red arrows), G8-/MyoD+ cells (green arrows) and G8+/MyoD+ cells (white arrows). Bar = 9 μM.

Tumors with properties of skeletal muscle were screened for the presence of Myo/Nog-like cells by double labeling with antibodies to G8 and noggin. Cells positive for both molecules were observed in a minimum of 80% of patients with ARMS, ERMS and spindle cell RMS (SCRMS), and 34% of patients with Wilms tumors (Table 3, Fig 4). Fifty percent or greater of RMS tissue sections and 40% of Wilms sections contained G8+/noggin+ cells (Table 3). Cells labeled for G8 or noggin alone cells were extremely rare. The distribution and prevalence of G8+/noggin+ cells varied between RMS and Wilms tissue sections from different patients. In some sections, Myo/Nog-like cells were present in one or multiple small clusters containing 2–6 cells, whereas in other sections, the core contained an abundance of double labeled cells (Fig 5). Only one G8+/noggin+ cell was found in 11% of sections of normal skeletal muscle (Table 3). Normal kidney tissue contained a few Myo/Nog cells per section (Table 3).

**Table 3:** RMS and Wilms tumors contain cells immunoreactive for both G8 and noggin.

Tissue	# of Subjects	% Subjects with G8+/Nog+ cells	# Sections	% Sections with G8+/Nog+ cells
**ARMS**	**5**	**80**	**11**	**73**
**ERMS**	**5**	**80**	**10**	**70**
**PRMS**	**9**	**88**	**19**	**63**
**SCRMS**	**2**	**100**	**4**	**50**
**Sk Muscle**	**3**	**33**	**18**	**11**
**Wilms**	**24**	**34**	**25**	**40**
**Kidney**	**2**	**100**	**3**	**100**

Tissue sections from tumors and normal skeletal muscle (Sk Muscle) and kidney were double labeled with antibodies to G8 and noggin (Nog), and species specific fluorescent secondary antibodies. % subjects and sections with G8+/noggin+ cells = (number of subjects and sections with double labeled cells ÷ total subjects and sections) X 100. G8+/noggin+ cells were found in the majority of subjects’ tumors and tissue sections. Labeled cells were also present in normal skeletal muscle and kidney. Statistical significance between tumor types was found within the group of subjects containing G8+/noggin+ cells (Chi-square, p = 0.01). Pairwise comparisons using the Fisher exact test revealed a significant difference between the number of PRMS and Wilms subjects containing G8+/noggin+ cells (p = 0.007).

Identity of Myo/Nog-like cells in RMS and Wilms tumors as skeletal muscle precursors was confirmed by co-localization antibodies to G8 and the skeletal muscle specific transcription factor MyoD (Fig 5). Sections from all tumor types contained subpopulations of G8+/MyoD+ cells. G8+/MyoD-negative (-) and G8-/MyoD+ cells also were present among the double labeled cells. Some cells were negative for both markers. These results are consistent with our previous findings indicating that MyoD mRNA is not translated in all G8+/noggin+ cells [1, 7, 12, 46]. It is also likely that MyoD is downregulated in the more differentiated tumor cells.

### Antibodies to G8 and filensin and a probe for filensin mRNA co-localize in RMS and Wilms tumors

**Fig 6:**
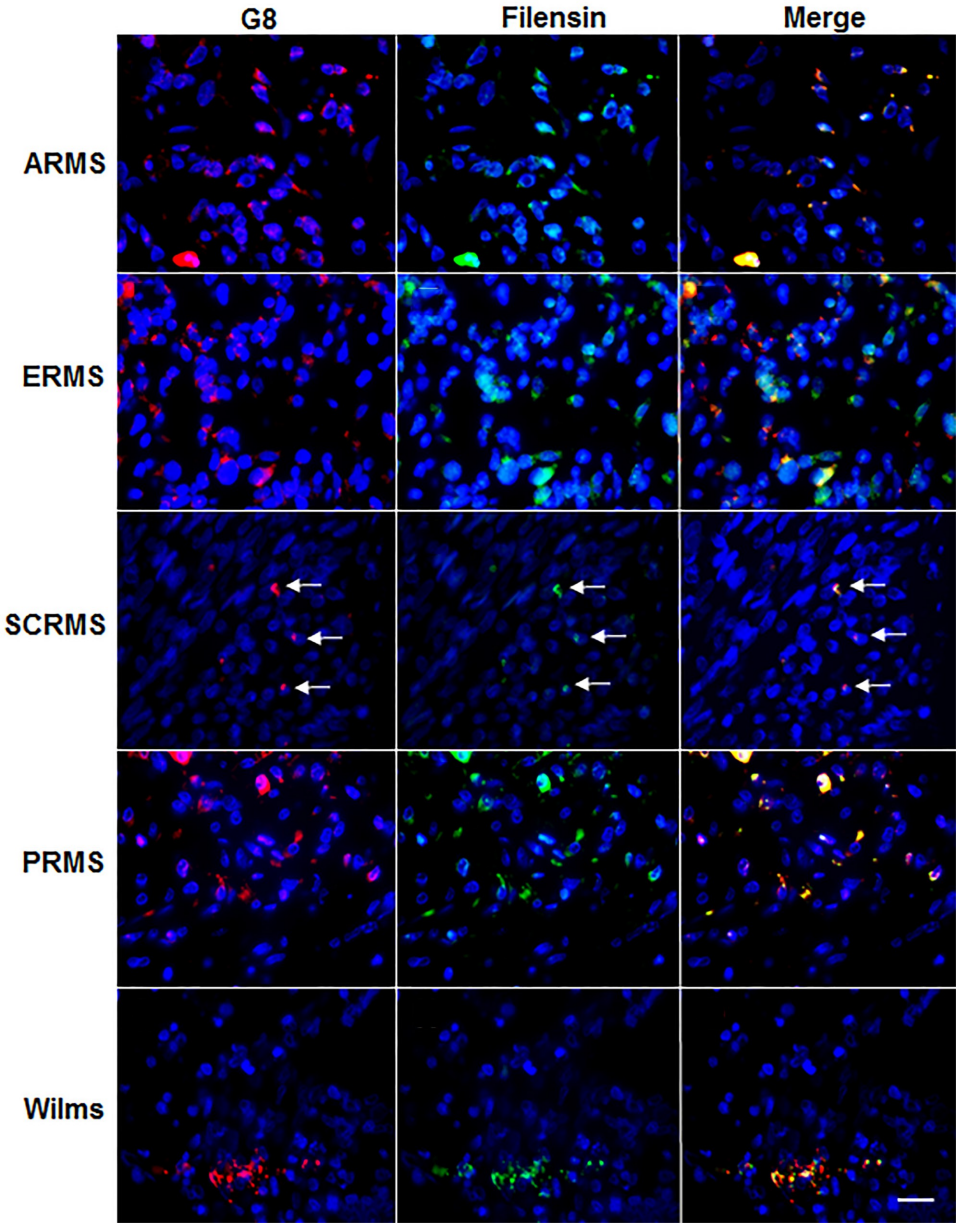
Co-localization of the G8 mAb and filensin polyclonal antiserum in human RMS and Wilms tumor tissue sections. Sections of human ARMS of the nose, ERMS from an intrabdominal tumor, spindle cell RMS (SCRMS) from the testis, pleomorphic RMS (PRMS) of the retroperitoneum and Wilms (kidney) tumors were double labeled with the G8 mAb (red) and polyclonal antibodies to filensin (green). Nuclei were stained with Hoechst dye (blue). Overlap of red and green appears yellow in merged images. RMS and Wilms tumor sections contained subpopulations of cells double labeled with antibodies to G8 and filensin. Bar = 9 μM.

Myo/Nog-like cells in myogenic tumors were screened for BF immunoreactivity by double labeling with the G8 mAb and a polyclonal antiserum to filensin. Cells labeled for both G8 and filensin were found in greater than 80% of patients’ tumors and 63% of sections from ARMS, ERMS, SCRMS and PRMS (Table 4; Fig 6). A few G8+/filensin- and G8-/filensin+ cells were present in three and seven percent of ERMS cores, respectively. No single labeled cells were observed in ARMS cores. Half of the patients with Wilms tumors and one third of the sections contained G8+/filensin+ cells (Table 4; Fig 6). As was the case with sections double labeled for G8 and noggin, the G8+/filensin+ cells were a subpopulation within the tissue. Some sections contained small clusters of these cells while others had larger portions of the core filled with fluorescent cells (Fig 6). Although G8+ cells were present in normal skeletal muscle and kidney, staining with the filensin antibody was not detected (Table 4).

**Fig 7:**
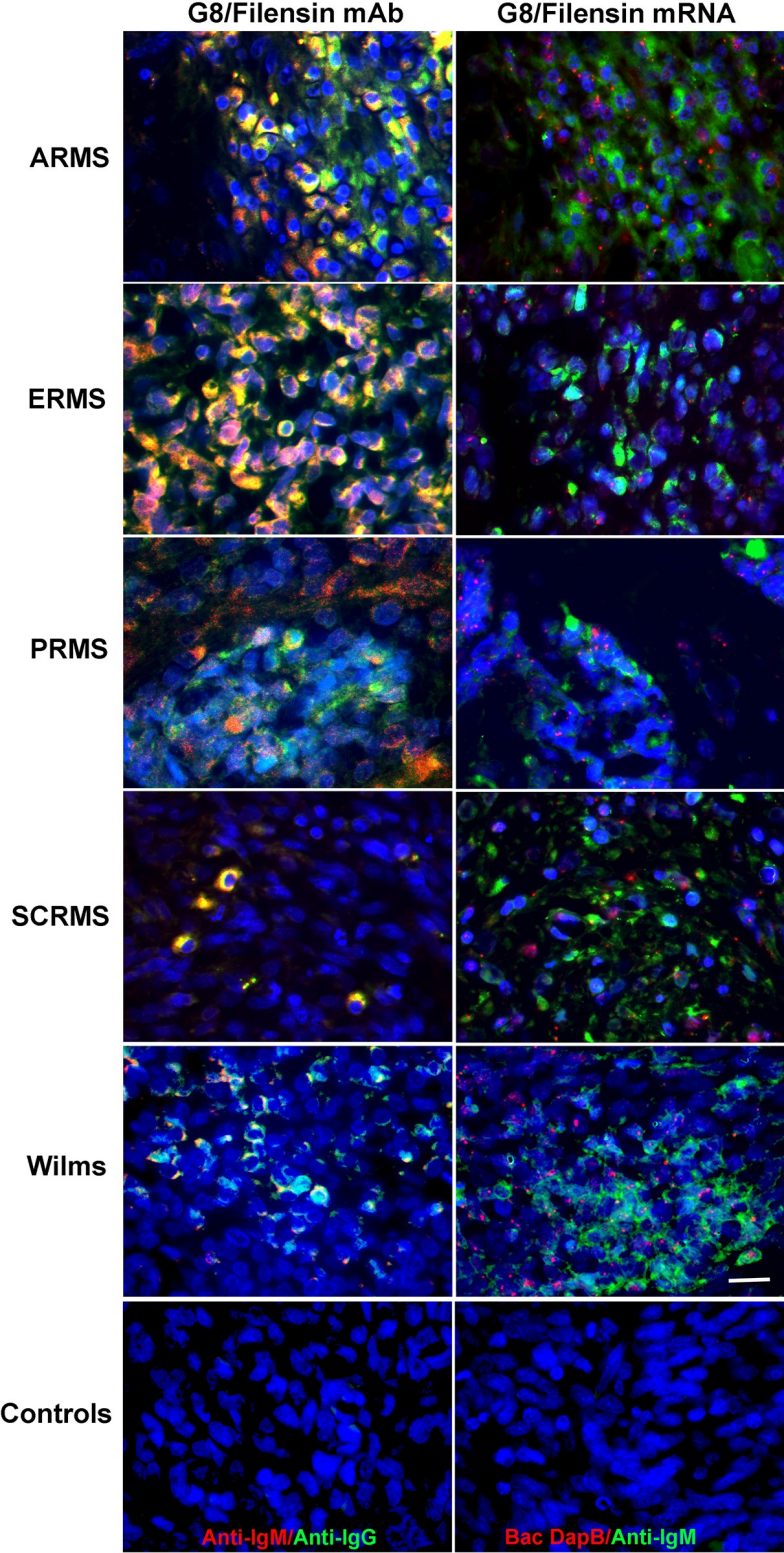
Co-localization of G8 with the filensin mAb and filensin mRNA in human RMS and Wilms tumor tissue sections. Tissue sections were double labeled with G8 (red) and the 1C1 filensin mAb (green), and G8 (green) and an anti-sense probe to filensin mRNA (red). Overlap of red and green appears yellow in merged images. G8 co-localized with the filensin mAb and filensin mRNA. Bar = 9 μM.

The specificity of the filensin polyclonal antiserum was verified with a mAb to filensin (Fig 7). Further confirmation that Myo/Nog-like cells expressed filensin was obtained by *in situ* hybridization with co-localization of the probe for filensin mRNA and the G8 mAb. Incubation in secondary antibodies alone and an in situ hybridization probe for bacteria DapB mRNA produced a weak, diffuse hue over the tissue (Fig 7).

**Table 4:** RMS and Wilms tumors contain cells immunoreactive for both G8 and filensin.

Tissue	# of Subjects	% Subjects with G8+/Fil+ cells	# Sections	% Sections with G8+/Fil+ cells
**ARMS**	**7**	**100**	**11**	**73**
**ERMS**	**23**	**83**	**58**	**64**
**PRMS**	**3**	**100**	**5**	**100**
**SCRMS**	**2**	**100**	**3**	**100**
**Sk Muscle**	**2**	**0**	**4**	**0**
**Wilms**	**5**	**50**	**13**	**33**
**Kidney**	**1**	**0**	**1**	**0**

Tissue sections from alveolar RMS (ARMS), embryonal RMS (ERMS), spindle cell RMS (SCRMS), pleomorphic RMS (PRMS) and Wilms tumors, and normal skeletal muscle (Sk Muscle) and kidney were double labeled with the G8 mAb and polyclonal antibodies to filensin (Fil) and species specific fluorescent secondary antibodies. % subjects and sections with G8+/filensin+ cells = (number of subjects and sections with double labeled cells ÷ total subjects and sections) X 100. G8+/filensin+ cells were found in the majority of subjects’ RMS tumors and tissue sections but were not observed in normal skeletal muscle or kidney sections. No significant difference between tumor types was found in the number of subjects containing G8+/filensin+ cells (Chi-square, p = 0.11).

### Antibodies to BF proteins co-localize in cells of RMS and Wilms tumors

**Fig 8:**
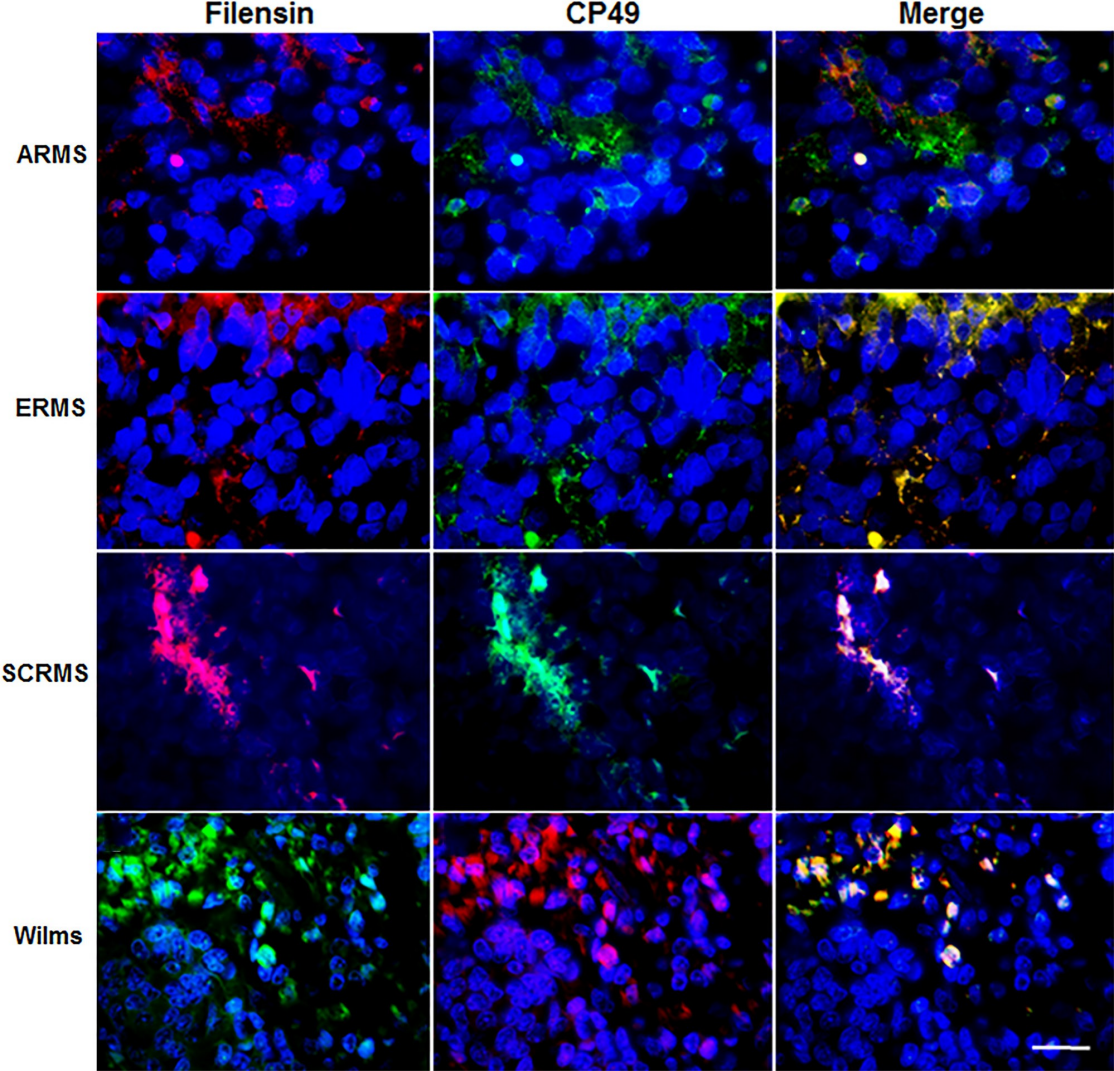
Co-localization of antibodies to filensin and CP49 in RMS and Wilms tumor tissue sections. Sections of human ARMS of the forearm, ERMS from the temporal region, spindle cell RMS (SCRMS) of the thigh and Wilms (kidney) tumors were double labeled with antibodies to filensin (red) and CP49 (green). Nuclei were stained with Hoechst dye (blue). Overlap of red and green appears yellow in merged images. RMS and Wilms tumors contained subpopulations of cells double labeled with antibodies to filensin and CP49. Bar = 9 μM.

A polyclonal antiserum to CP49 and mAb to filensin were applied to examine potential co-expression of the two BF proteins in tumors. Greater than 80% of ARMS, ERMS, SCRMS and Wilms patients, and 72% of sections from these tumors contained cells double labeled for CP49 and filensin (Table 5; Fig 8). A few filensin+/CP49- and filensin-/CP49+ cells were observed in one section of ERMS, Wilms and SCRMS tumors. Neither CP49+ nor filensin+ cells were detected in sections of normal skeletal muscle or kidney (Table 5).

**Table 5:** RMS and Wilms tumors contain cells immunoreactive for both filensin and CP49.

Tissue	# of Subjects	% Subjects with Fil+/CP49+ cells	# Sections	% Sections with Fil+/CP49+ cells
**ARMS**	**35**	**91**	**66**	**81**
**ERMS**	**31**	**80**	**71**	**75**
**SCRMS**	**11**	**91**	**20**	**75**
**Skeletal Muscle**	**2**	**0**	**6**	**0**
**Wilms**	**10**	**90**	**15**	**73**
**Kidney**	**2**	**0**	**3**	**0**

Tissue sections from tumors and normal skeletal muscle and kidney were double labeled with antibodies to filensin (Fil) and CP49 and species specific fluorescent secondary antibodies. % subjects and sections with filensin+/CP49+ cells = (number of subjects and sections with double labeled cells ÷ total subjects and sections) X 100. Filensin+/CP49+ cells were found in the majority of subjects’ ERMS, ARMS, SCRMS and Wilms tumors and tissue sections. Neither filensin nor CP49 antibody staining was observed in normal skeletal muscle or kidney. No significant difference between tumor types was found in the number of subjects with filensin+/CP49+ cells (Chi-square, p = 0.58).

### Antibodies to G8 and noggin, but not BFs, bind to a subpopulation of cells in leiomyosarcomas

**Fig 9:**
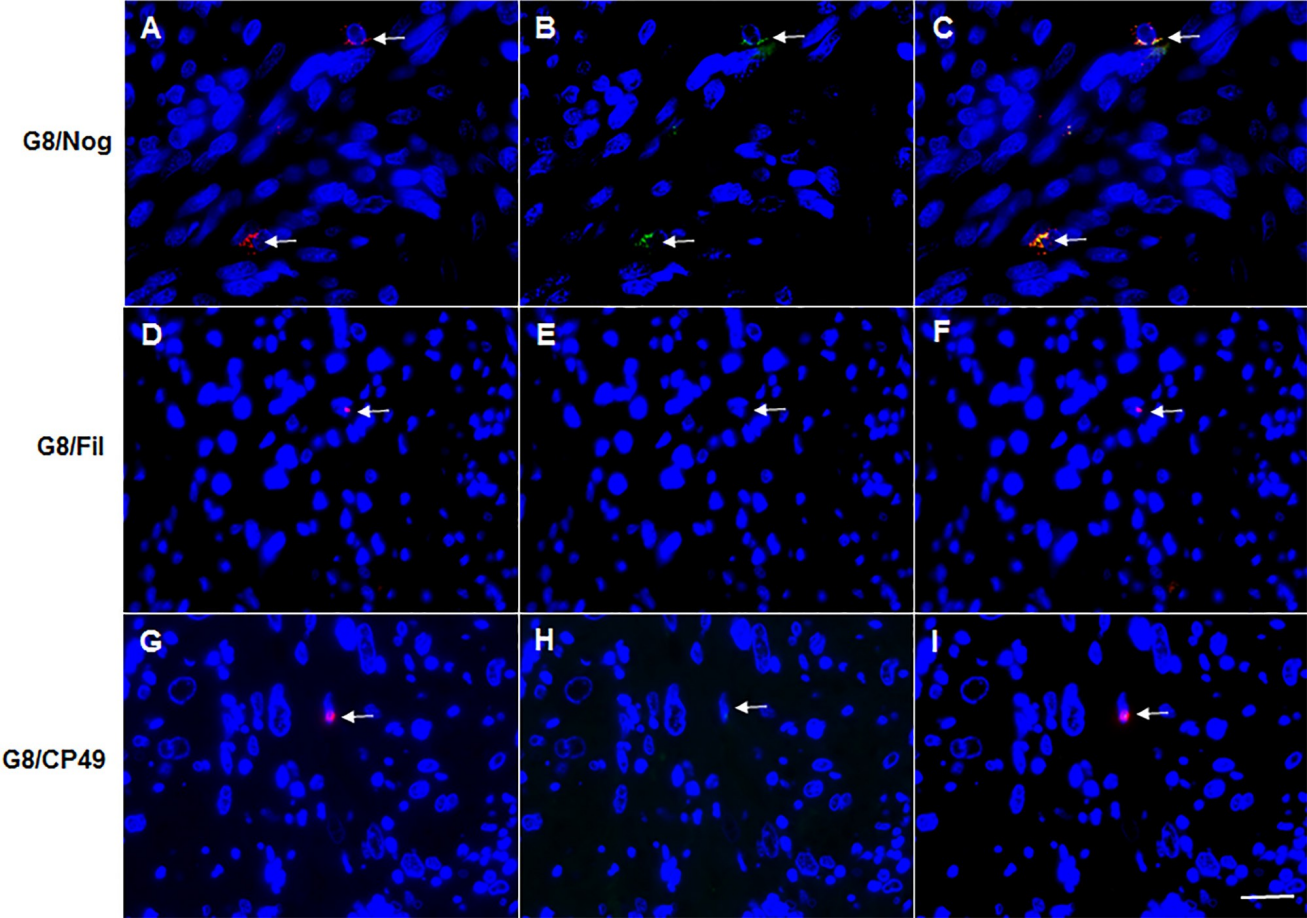
Antibodies to G8 and noggin, but not BF proteins, label cells in leiomyosarcomas. Sections of leiomyosarcoma tumor tissue obtained from fibrous tissue (A-C) and the thigh (D-I) were double labeled with antibodies to G8 and noggin, G8 and filensin (Fil) or G8 and CP49. Nuclei were stained with Hoechst dye. Overlap of red and green appears yellow in merged images. Antibodies to G8 and noggin are co-localized in leiomyosarcomas. Antibodies to BF proteins did not label cells in large leiomyosarcoma tissue sections. Bar = 9 μM.

Thirty nine percent of subjects with leiomyosarcoma and approximately one third of the sections from these tumors of smooth muscle origin contained small numbers of G8+/noggin+ cells (Table 6, Fig 9). All sections of leiomyosarcomas contained two to nine G8+ cells. Neither filensin nor CP49 was detected in G8+ or G8- cells in these tumor sections (Table 6; Fig 9).

**Table 6:** Antibodies to Myo/Nog cell markers, but not BF proteins, label cells in human leiomyosarcomas.

**# Subjects**	**% Subjects with G8+/Nog+ cells**	**# Sections**	**% Sections with G8+/Nog+ cells**
**19**	**39**	**36**	**33**
**# Subjects**	**% Subjects with G8+/Fil+ cells**	**# Sections**	**% Sections with G8+/Fil+ cells**
**5**	**0**	**6**	**0**
**# Subjects**	**% Subjects with G8+/CP49+ cells**	**# Sections**	**% Sections with G8+/CP49+ cells**
**4**	**0**	**5**	**0**
**# Subjects**	**% Subjects with Fil+/CP49+ cells**	**# Sections**	**% Subjects with Fil+/CP49+ cells**
**2**	**0**	**4**	**0**

Tissue sections from leiomyosarcoma tumors were double labeled with antibodies to the G8 mAb and polyclonal antibodies to noggin (Nog), filensin (Fil) and Cp49, and filensin and CP49. Four of five sections double labeled for G8 and filensin, and three of four sections labeled with G8 and CP49 were greater than 7 mm. The remaining sections were tissue array cores. Antibody binding was visualized with species and subclass specific fluorescent secondary antibodies. % subjects and sections with +/+ cells = (number of subjects and sections with double labeled cells ÷ total subjects and sections) X 100. Although Myo/Nog-like cells were present in approximately one third of leiomyosarcoma tissue sections, antibodies to filensin and CP49 were not detected.

### G8+ cells in skin carcinoma and malignant melanoma tumor sections do not label with antibodies to filensin and CP49

Our previous work demonstrated the presence of Myo/Nog-like cells in human and mouse skin tumors [47]. Skin carcinomas and malignant melanomas were screened for beaded filament immunoreactivity. As expected, squamous and basal carcinoma and malignant melanoma tumor sections contained G8+ cells (Table 7). Syringocarcinomas also contained G8+ cells (Table 7). No G8+ or G8- cells in these tumor sections bound antibodies to filensin or CP49 (Table 7).

**Table 7:** Antibodies to G8, but not filensin or CP49, label cells in human skin tumors.

Tissue	# of Subjects/ # of Sections	% Sections with G8+ cells	% Sections with Fil+ cells	% Sections with CP49+ cells
**Squam Cell Car**	**3/6**	**100**	**0**	**0**
**Basal Cell Car**	**3/6**	**100**	**0**	**0**
**Malig Mel**	**6/19**	**75**	**0**	**0**
**Syringocar**	**3/6**	**67**	**0**	**0**

Tissue sections from squamous (squam) and basal cell carcinomas (car), malignant melanomas (malig mel) and syrigocarcinomas (syringocar) were double labeled with the G8 mAb and polyclonal antibodies to filensin and CP49. Antibody binding was visualized with species specific fluorescent secondary antibodies. % sections with G8+, filensin (FIl)+ and Cp49+ cells = (number of sections with labeled cells ÷ total sections) X 100. Although G8+ cells were present in greater than two thirds of the sections, filensin and CP49 were not detected.

## Discussion

Our studies of chick embryo development led to the discovery of a novel subpopulation, called Myo/Nog cells, that are integrated from the epiblast into all three germ layers and are later found in fully mature fetal organs, including those lacking skeletal muscle [2, 3, 6]. In addition to their critical role as modulators of BMP signaling, Myo/Nog cells can fuse to form multinucleated, skeletal myofibers *in vitro* or develop into myofibroblasts in response to wounding [2, 3, 7–9, 48]. The ability of Myo/Nog cells to synthesize contractile proteins reflects their expression of MyoD and stable commitment to the skeletal muscle lineage regardless of their environment [1, 2, 5, 6].

Within the human lens, Myo/Nog cells bind antibodies to BF proteins that are considered specific markers of the lens fiber cell lineage [28, 34, 44]. Labeling of Myo/Nog cells with antibodies to BF proteins was unexpected because lens epithelial and Myo/Nog cells have different embryological origins [1–4, 49]. To our knowledge, the presence of BF proteins in tissues outside of the lens during development, including the extraocular muscles, has not been reported.

Immunoreactivity for BF proteins also was detected in human RMS cell lines. While additional experiments are required to identify the specific epitopes of BF antibodies in Myo/Nog and RMS cells, the filensin and CP49 antibodies produced bands of similar molecular weights in Western blots of RMS and lens extracts. Therefore, synthesis of BF proteins may be a characteristic of the lens epithelial lineage, Myo/Nog cells within the lens and subpopulations of RMS cells. It is presently unknown whether the significantly different percentages of G8+/noggin+/BF+ cells in RC13 and RD RMS cells *in vitro* reflects the aggressiveness of ARMS and ERMS tumors *in vivo*.

Subpopulations of Myo/Nog-like cells expressing G8 and noggin also were identified in human RMS, Wilms and leisomyosarcoma tumors. The number of G8+/noggin+ cells varied between patients; however, in all cases, they were a small subpopulation within tissue sections. Whereas practically all G8+ cells contained detectable levels of BF antibodies in RMS and Wilms tumor sections, BFs were not detected within or outside Myo/Nog-like cells in leiomyosarcomas or skin tumors. More extensive analyses of non-myogenic sarcomas, carcinomas and tumors with a mixed phenotype are required to determine whether immunoreactivity for filensin and CP49 is, in fact, diagnostic of tumors with properties of skeletal muscle.

The appearance of BF+, Myo/Nog-like cells in RMS and Wilms tumors may be a manifestation of an environment with features of both skeletal muscle and wounds. While Myo/Nog cells were extremely rare in normal skeletal muscle tissue sections screened in this study, comprehensive analyses of BF expression in diseased and regenerating muscles are warranted given that Myo/Nog cells home to wounds and differentiate into myofibroblasts that are immunoreactive for BFs in the lens [7, 8, 12].

One possible explanation for co-localization of antibodies to G8, noggin, filensin and CP49 in only a subpopulation of cells in RMS tumors is that they are transformed stem cells whose progeny downregulate expression of all four molecules. Cells that give rise to RMS tumors have not been identified. Candidates for RMS initiating/stem cells include the side population (SP) cells, mesenchymal stem cells, satellite cells, myoblasts, dedifferentiating myocytes and endothelial progenitor cells [14, 50–55]. The Myo/Nog cell is a logical candidate for a source of RMS tumors that often arise outside of skeletal muscle because they are widely distributed in embryonic and adult tissues and are inherently myogenic [2, 5–7, 10–12]. Testing the potential of G8+ RMS cells to give rise to tumors and the effects of depleting G8+/noggin+/BF+ cells on tumor expansion will be required before assigning them a pathogenic role in RMS.

A counter argument to BF expression coincident with Myo/Nog cell transformation is that antibodies to filensin and CP49 bind G8+ cells in the lens. Tumors in the lens are rare in animals and have not reported in humans [56–58]. Lens malignancies in animals are correlated with injury [58, 59]. While only one out of nine feline tumors was immunoreactive for the lens protein crystallin alpha A, all were stained with an antibody to vimentin, and some were also positive for muscle specific actin and desmin [60]. Screening lens tumors for Myo/Nog cell markers, MyoD family members and contractile proteins may reveal a potential source of these cancers since the Myo/Nog cell and its derivatives are the only cells in the lens that synthesize skeletal muscle specific proteins [7].

Finally, undifferentiated sarcomas represent a diagnostic and treatment challenge, in part because markers for identifying a particular lineage of origin are often absent. Screening these tumors for the presence of Myo/Nog cell markers and BF proteins may allow for subclassification of these malignancies.
